# Malignant peripheral nerve sheath tumour—A case report

**DOI:** 10.1016/j.ijscr.2019.10.004

**Published:** 2019-10-10

**Authors:** Senthilkumar A.C., S. Sridharan, B. Mahendra, V. Chander

**Affiliations:** a Department of Surgical Oncology, Saveetha Medical College Hospital, India; b Department of General Surgery, Saveetha Medical College Hospital, India; c Department of Pathology, Saveetha Medical College Hospital, India

**Keywords:** Malignant, Peripheral, Nerve, Sheath, Tumour, Pre-sacral, MPNST, Case, Report

## Abstract

**Introduction:**

Malignant peripheral nerve sheath tumors (MPNST) are rare, aggressive sarcomatous tumors that arise from peripheral nerve sheaths and show schwann cell differentiation (Thangadurai, 2017) [1]. They commonly arise among patients with neurofibromatosis I and following radiation therapy (Farid et al., 2014). High resolution PET scans confirm the diagnosis and the definitive treatment is surgical excision. Post operative radiotherapy plays an important role in disease free survival rates. Chemotherapy is offered to people who have a systemic disease.

**Presentation of case:**

This case report is on a 47 year old lady, a known case of Von Recklinghausen, who complained of a vague mass in the pelvis with recent history of pain to her right thigh. Pelvic examination revealed a vague mass on the right side of her pelvis. She was planned for excision of the mass. Intraoperative findings were consistent with the clinical and radiological findings. She is currently on regular follow-up and disease free.

**Discussion:**

MPNSTs are rare tumours that arise in patients with neurofibromatosis I. Mutations at the genetic level is responsible for the development of MPNST. Early presentation, radiological confirmation, aggressive margin free surgery followed by chemoradiation helps in near complete cure of the disease.

**Conclusion:**

MPNST are difficult to manage because of their aggressive nature and the limitations in early diagnosis and management. In patients with Von Recklinghausens disease, malignancy must be suspected when a patient presents with the complaints as mentioned in this case report.

## Highlights

• Pre-sacral mass presenting with right thigh shooting pain in the setting of Von Recklinghausen.
• Radiological imaging was crucial in providing a road map pre-op.
• Mass abutting the internal iliac artery and possibly pressing on the sacral nerve roots.

## 1 Case presentation

A middle aged lady walked in to the out patients department with complaints of right sided pelvic shooting type pain for 2 months duration. The pain radiated to her right upper thigh for the last 10 days. She was a known case of Von Recklinghausen syndrome and had multiple cutaneous neurofibromas all over her body with the classical café au lait spots over her back and abdomen. She had no co-morbid illness, no relevant surgical history and no family history of neurofibromatosis.

After obtaining consent from the patient, on examination her general condition and vitals were stable. Abdominal examination was insignificant as were her cardiovascular and respiratory system examination. Pelvic examination revealed a vague mass on the right side of her pelvis which was pushing the rectum to the left side.

**Figs. 1 and 2:**
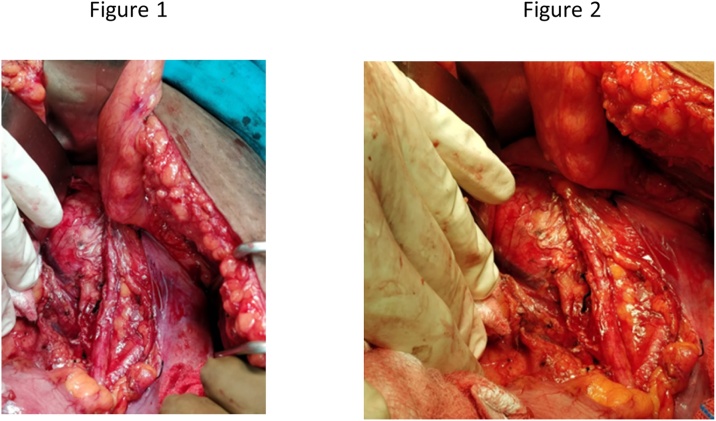
Shows an intro-op photo of the pre-sacral MPNST.

**Figs. 3 and 4:**
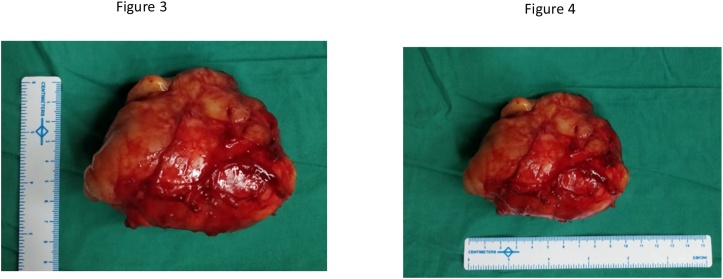
Shows the dimensions of the resected specimen 9 × 7.5 cm).

Ultrasonogram of the abdomen and pelvis was normal. Contrast enhanced CT of the abdomen and pelvis revealed a 6 × 7 cm mass in the infragluteal region abutting the sciatic nerve and displacing the internal iliac artery medially. High resolution CT chest was normal. After obtaining anaesthetic fitness, she was posted for pelvic mass excision under general anaesthesia. The surgical oncology team, with more than 15 years of experience in Saveetha Medical College and Hospital performed the procedure. Prophylactic 3^rd^ generation cephalosporin was given intravenously prior to surgery. Patient was in lithotomy position and a lower laparotomy incision was made. Intra-operatively, 10 × 8 cm firm, solid encapsulated mass was seen arising from the right lateral pelvic wall (Figs. 1 and 2). Figs. 3 and 4 shows the resected specimen. Nodular chains of pelvic lymph nodes were seen and removed along with the mass. Approximately 300 ml blood was lost during the 2.5 h of surgery. Post operatively DVT prophylaxis was advised along with intravenous antibiotics and painkillers. Oral diet started on post operative day 1. She was mobilized on post operative day 4. She experienced relief of her symptoms and was discharged on post operative day 6. She is under regular follow-up (every 6 months) and doing well.

**Fig. 5:**
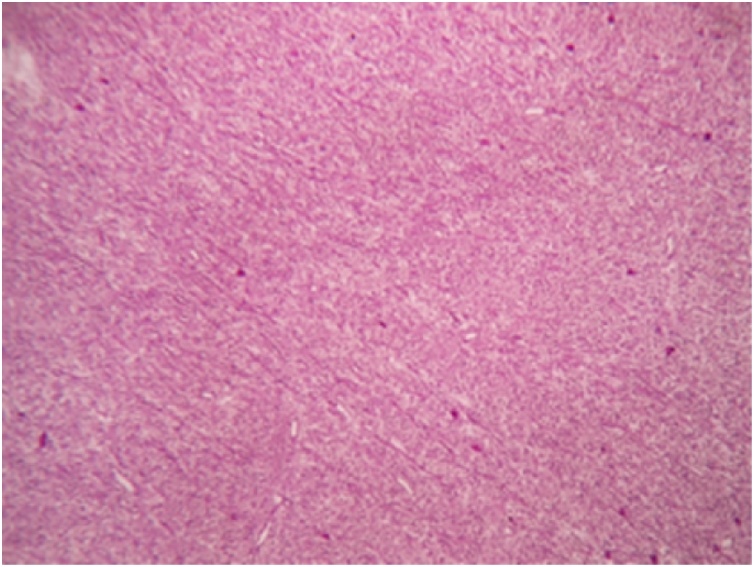
Shows a neoplasm composed of delicate spindle shaped cells arranged in bundles and fascicles (H&E ×40).

**Fig. 6:**
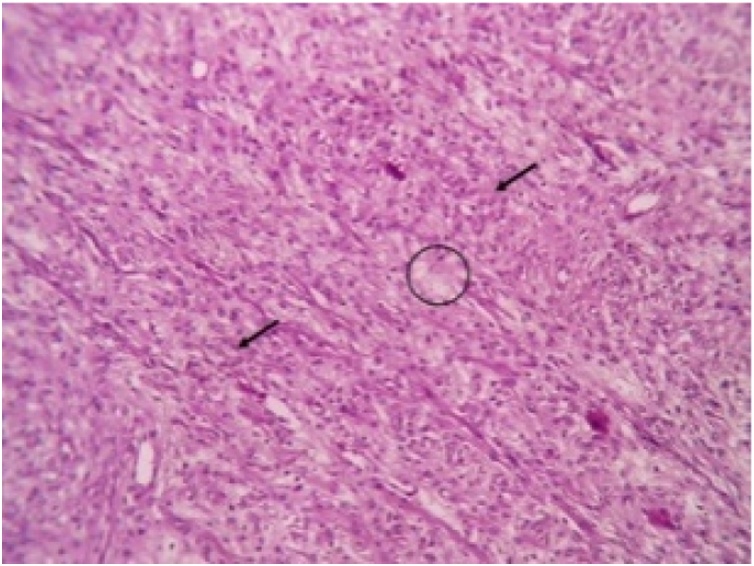
Shows the tumor cells (arrow) show elongated wavy nuclei and eosinophilic cytoplasm. The cells are arranged haphazardly in a fibromyxoid stroma (circle) (H&E ×100).

**Fig. 7:**
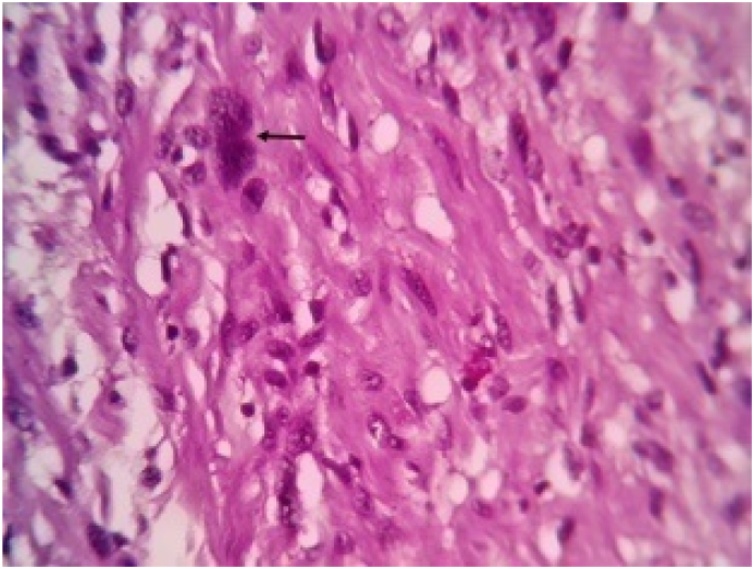
shows tumor cells with plump elongated nuclei and moderate eosinophilic cytoplasm (arrow) (H&E ×400).

**Fig. 8:**
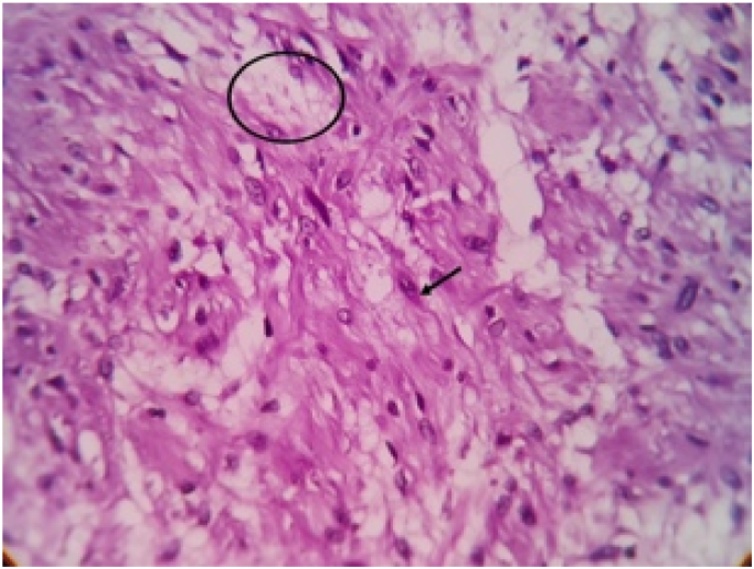
Shows focal areas showing rhabdoid differentiation - Strap cells (Arrow). Focal foamy macrophages are also seen (Circle) (H&E ×400).

**Fig. 9:**
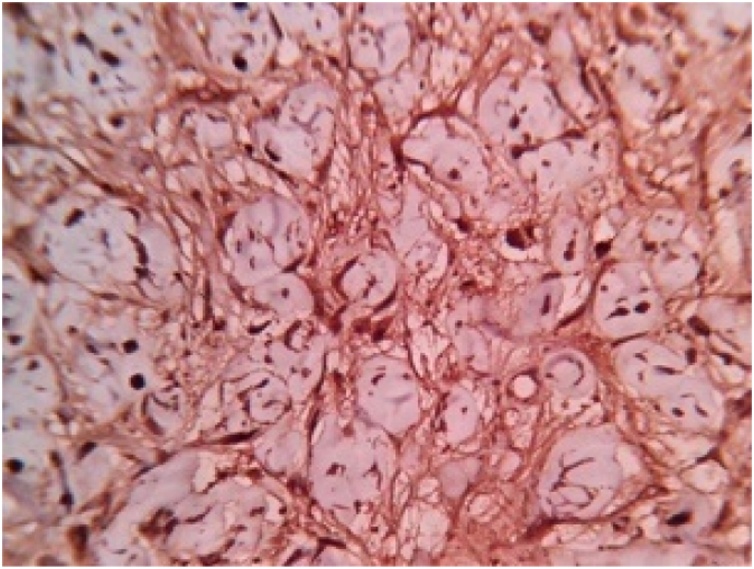
Shows S100 immunostaining showing strong nuclear and cytoplasmic positivity in 95% of the tumor cells (S100 IHC ×400).

**Fig. 10:**
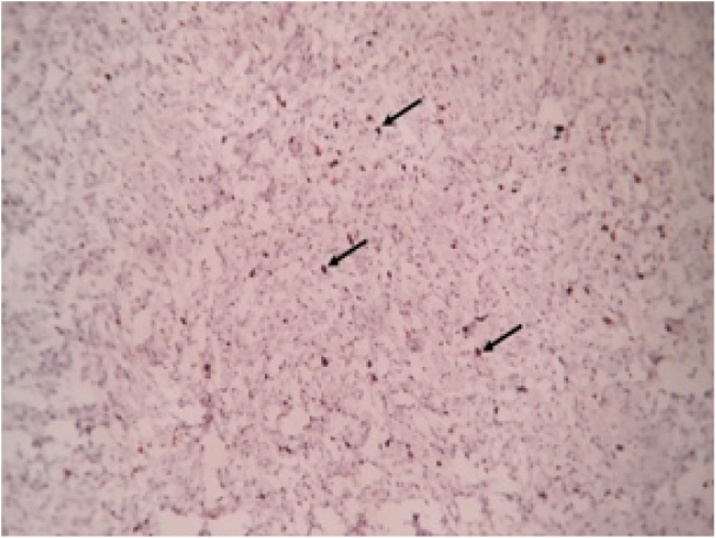
Shows Ki67 immunostaining showing Strong nuclear positivity in 10–15% of the tumor cells (arrows) (Ki67 IHC ×100).

Histopathological report revealed features suggestive of low grade malignant peripheral nerve sheath tumor. Microscopically, the mass showed spindle shaped cells arranged in bundles and fascicles with wavy nuclei in an eosinophilic cytoplasm. There were areas that showed rhabdoid differentiation with skeletal muscle fibres, myofibroblasts and few rhabdomyoblasts (Fig. 5, Fig. 6, Fig. 7, Fig. 8). The tumor stained positive for S-100 and Ki67 on immunohistochemistry (Fig. 9, Fig. 10).

## 2 Introduction

Malignant peripheral nerve sheath tumors (MPNST) are classified as sarcomas that arise from peripheral nerves, a pre-existing peripheral nerve sheath tumour that has undergone differentiation or in concurrence with neurofibromatosis type I (NF I) syndrome [3]. NF 1 syndrome is characterized by the loss of the tumour suppressor gene, neurofibromin, and clinically the patient presents with multiple plexiform neurofibromas all over the body. The standard of care is an R0 resection with adjuvant radiotherapy. This case report has been reported in line with the SCARE criteria [4].

## 3 Epidemiology/etiology/pathogenesis

MPNST has no gender predilection and commonly occurs between the 3^rd^ and 6^th^ decade of life [5]. Von Recklinghausen first described this condition in the late 1880s. It is characterized by the presence of café au lait spots (cutaneous hyperpigmentation) and multiple plexiform neurofibromas, which are slow growing peripheral nerve sheath tumours with a potential to rapidly increase in size, turn malignant and produce a mass effect. NF1 also presents with axillary freckling, optic gliomas, iris hamartomas termed Lisch nodules, bone dysplasia, cardiovascular abnormalities, and other malignancies (GIST, rhabdomyosarcoma, AML) [2].

## 4 Clinical presentation and diagnosis

The lifetime risk of a patient with neurofibromatosis I developing MPNST is 8%–13% [6]. A rapidly enlarging palpable mass in a previously asymptomatic patient with NF1 should raise the suspicion of malignancy. Pain, paraesthesia, weakness and other neurological deficits are common complaints the patient presents with. The most common site affected are the sciatic nerve roots. A size of more than 5 cm requires surgery because of its malignant potential and risk of metastasis to the lung. Pleural and bone metastasis is rare [2].

MRI is the imaging modality of choice. Tumours > 5 cm, invasion of fat planes, heterogeneity, ill-defined margins and surrounding edema are highly suggestive of MPNST [7,8]. HRCT chest and a preoperative bone scan is required as a part of the metastatic work-up. FDG-PET works by assessing the intracellular glucose levels [9] in highly metabolic tumour cells.

Fine needle aspiration and core needle biopsies play an important role in staging the disease. FNA is preferred in cases where a recurrence is suspected.

## 5 Treatment

Complete surgical resection and achievement of tumour free margins is the mainstay of treatment. If the tumour size is more than 5 cm, neoadjuvant radiotherapy is advocated to shrink the size of the tumour and reduce local recurrence [10].

Radiotherapy yields good results with respect to improved survival rates. Preoperative radiotherapy significantly reduces the size of the tumour. This approach reduces the total dose of radiation required and better tumour localization. Radiation causes tumour necrosis and reduces the chances of tumour spill thereby making limb salve surgery successful [11]. Post operative radiation requires higher doses of radiation, interferes with wound healingand the potential risk of seeding the surgical scar with malignant cells.

Chemotherapy is preferred when either the disease is too small to detect or diffuse and is implicated for use in high grade and metastatic disease.

## 6 Conclusion

MPNST are difficult to manage because of their aggressive nature and the limitations in early diagnosis and management. Advances in computed tomographic scans and PET scans coupled with expert immunohistochemical analysis of lesions can accurately identify the stage of the disease and can predict its aggressive nature. Molecular targeted therapies following surgery for MPNST should be developed to render a patient disease free. In patients with Von Recklinghausens disease, malignancy must be suspected when a patient presents with the complaints as mentioned in this case report.

## Sources of funding

No external sources of funding. This case report was self funded.

## Ethical approval

Informed consent was obtained from the patient for publication of this case report.

## Consent

Written informed consent was obtained from the patient for publication of this case report and accompanying images. A copy of the written consent is available for review by the Editor-in-Chief of this journal on request.

## Author’s contribution

Dr. A. C. Senthilkumar: Chief surgeon who operated on this case.

Dr. Balu Mahendra: 1^st^ assistant.

Dr. Sivaram Sridharan: 2^nd^ assistant, writing the paper.

Dr. Vimal Chander: interpretation of the gross and microscopic pathological slides.

## Registration of research studies

Not applicable.

This is a case report.

## Guarantor

Dr. A. C. Senthilkumar.

## Provenance and peer review

Not commissioned, externally peer-reviewed.

## Declaration of Competing Interest

No financial/personal relationships/conflicts of interest.
